# Bleeding in Locally Invasive Pelvic Malignancies: Is Hypofractionated Radiation Therapy a Safe and Effective Non-Invasive Option for Securing Hemostasis? A Single Institution Perspective

**DOI:** 10.7759/cureus.2137

**Published:** 2018-02-02

**Authors:** Muhammad Shuja, Saadiya Nazli, Muhammad Atif Mansha, Asif Iqbal, Reham Mohamed, Mutahir A Tunio, Eyad F Alsaeed, Mushabbab Asiri, Yasser Bayoumi

**Affiliations:** 1 Department of Radiation Oncology, King Fahad Medical City, Riyadh, Saudi Arabia; 2 Department of Transfusion Medicine, King Khalid University Hospital, King Saud University - Medical City, Riyadh, Saudi Arabia; 3 Section of Radiation Oncology, Department of Oncology, Aga Khan University Hospital, Karachi, Pakistan; 4 Medical Physics Department, King Fahad Medical City, Riyadh, Saudi Arabia; 5 Department of Radiation Oncology, King Khalid University Hospital, King Saud University - Medical City, Riyadh, Saudi Arabia; 6 Radiation Oncology Department, King Fahad Medical City, Riyadh, Saudi Arabia

**Keywords:** radiation therapy, palliative, hypofractionated, bleeding, pelvic malignancy

## Abstract

Introduction: Control of bleeding due to locally invasive disease is of paramount importance in the management of cancer patients. This study was undertaken to explore the outcomes of palliative intent hypofractionated radiation therapy (HRT) in advanced stage pelvic malignancies that presented with bleeding.

Methods: This study enrolled patients treated with palliative intent hypofractionated radiation therapy from July 2015 to November 2017. In the inclusion criteria, all these patients had the common presenting complaint of bleeding from the tumor. These patients were not treated with radiation therapy before for the same indication. Patients with known bleeding disorders and those undergoing parallel interventions for bleeding control were excluded from the study. Bleeding was categorized based on the World Health Organization (WHO) scale for the classification of bleeding. Response assessment was classified into a complete response, partial response and no response. A comparison was made for the bleeding scale before and after HRT using the Wilcoxon signed rank test. The comparison of mean hemoglobin levels before and after the HRT was calculated by paired t-test.

Results: Forty-two patients with advanced pelvic malignancies qualified for inclusion in the study after applying the inclusion/exclusion criteria. Among those analyzed, the median age was 67 years (range 37 – 95 years). The male and female proportion was 38% and 62% respectively. Different cancers included uterine cancer 31%, cervical cancer 24%, bladder cancer 21%, rectal cancer 17% and vulvar cancer in 7%. The baseline bleeding scale in these cases was found to be grade 1 in 12%, grade 2 in 55% and grade 3 in 33% cases. The median dose in our cohort was 20 Gy in five fractions over one week (range was 8 Gy to 40 Gy). Following HRT, the WHO bleeding score at one month was recorded as grade 0 in 57%, grade 1 in 31%, grade 2 in 7%, grade 3 in 5% and grade 4 in none. Toxicity profile did not show any grade 3 or above acute toxicity in the study. Response rates were 57% complete response, 36% partial response and 7% no response. The mean hemoglobin level post-treatment versus pre-treatment was found to be 9.6 g/dL versus 7.3 g/dL.

Conclusions: Hypofractionated radiotherapy was found to be a safe and effective non-invasive palliative treatment modality for securing hemostasis in advanced pelvic malignancies that presented with bleeding.

## Introduction

Radiation therapy (RT) plays a significant role in the treatment of cancers either as a single definitive therapy or as a part of multimodality treatment. It is well learned from clinical practice that when tumors progress, the chances of bleeding increase with around 6-10% of advanced malignancies presenting with bleeding [1]. This may be in the form of epistaxis, hemoptysis, hematemesis, hematochezia, melena, vaginal bleeding, and or ulcerated skin lesions. This condition could lead to severe consequences when bleeding is unresponsive to conservative management, and the patient is unfit to undergo surgical intervention as in unresectable locally invasive tumors or poor performance status of the patient [2-3]. Sometimes the available options in the management of bleeding are known to give rise to significant complications [4-5]. Here the role of radiation therapy as a non-invasive local hemostatic agent becomes paramount [6-7]. Because if not adequately controlled, bleeding can impart significant impact on patient morbidity and mortality; and may even lead to death in uncontrolled cases [1].

Hemoglobin level of less than 6.5 g/dL is categorized as life-threatening, and blood transfusion is the only intervention option for patients who require immediate correction of anemia [2]. According to the guidelines published by the American Association of Blood Banks [8], transfusion is recommended for hospitalized patients without cardiovascular disease targeting the threshold of hemoglobin level of 7 to 8 g/dL and depending on their symptoms. Mechanism of action of radiation therapy as a hemostatic agent is attributable to its role in enhanced platelet adhesion to the extracellular matrix of human endothelial cells by an increase in the release of von Willebrand factor [9] while the long-term effects as a hemostatic agent could be explained by the vessel fibrosis combined with tumor remission [10].

In historical cohorts, radiation therapy has been used in various fractionation schedules to control bleeding (conventional and hypofractionated) [2-3, 6-7]. Based on these features, RT can prove to be a quick and effective treatment option in the palliative management of bleeding malignancies [1-3, 11-12]. Further, bleeding with advanced cancer is not only a significant source of distress to the patients and their families, but also results in poor quality of life, which is mainly attributed to prolonged hospitalization [2,11].

Hypofractionated radiotherapy in this setting could alleviate the sufferings and deliver a safe and effective palliative management modality for controlling symptomatic bleeding malignancies with minimal side effects or complications to preserve the quality of life (QoL) [11-12]. The role of hypofractionated RT as a non-invasive treatment option in advanced stage pelvic malignancies presenting with bleeding warrants further investigation due to the scarcity of literature in this subject area. This study was undertaken to explore the outcomes after hypofractionated radiation therapy (HRT) in advanced stage pelvic malignancies presenting with bleeding.

## Materials and methods

Study design

It was a prospective single arm, uncontrolled, single institutional study. This study enrolled all the cases of advanced pelvic malignancies presenting with bleeding that were treated with palliative intent hypofractionated radiation therapy at the Radiation Oncology Department of King Fahad Medical City (KFMC), Riyadh, Saudi Arabia from July 2015 to October 2017.

Inclusion and exclusion criteria

The inclusion criteria included advanced stage locally invasive pelvic malignancies, cases presenting with bleeding. The exclusion criteria included treatment with other hemostatic procedures (surgery, embolization, cautery, etc.), patients on continuous anticoagulation or other hematological parameter modifying agents, and those patients suffering from known congenital bleeding problems and hematological malignancies.

Objectives and tools

The primary objective of our study was to assess the efficacy of hypofractionated radiotherapy used with palliative intent to secure hemostasis in patients with locally invasive pelvic malignancies presenting with bleeding at KFMC radiation oncology department. The secondary objective of our study was to determine the safety of palliative intent hypofractionated radiotherapy. Our primary endpoint was bleeding at one-month post-HRT. The secondary endpoint was bleeding scale and toxicity at one-month post-HRT.

The bleeding scale was based on the World Health Organization (WHO) classification [13] with 0 = no bleeding, 1 = petechial bleeding, 2 = clinically significant bleeding, not requiring transfusion, 3 = bleeding requiring transfusion, and 4 = bleeding associated with fatality.

The response assessment was based on clinical exam and evaluation in the clinic at the time of follow-up. Complete response (CR) was defined as no bleeding at one-month post-HRT.  Partial response (PR) was a subjective decrease in bleeding at one-month post-HRT. No response (NR) was no change or increase in bleeding at one-month post-HRT. For further subset analysis, a satisfactory response was labeled if the bleeding at one-month post-HRT was either grade 0 or 1 based on WHO classification.

The toxicity was graded based on the common terminology criteria for adverse events (CTCAE) classification [14]. The European Cooperative Oncology Groups (ECOG) classification [15] was used for categorizing the performance status of patients. 0 = fully active, asymptomatic, 1 = symptomatic, fully ambulatory, 2 = symptomatic, in bed less than 50% of the day, 3 = symptomatic, in bed more than 50% of the day, but not bedridden, 4 = totally bedridden, and 5 = dead.

Data collection and statistical analysis

The study was initiated after formal approval from the institutional review board (IRB). Informed consent was obtained from the patients. Data was collected on questionnaires and then uploaded onto a common database.  The main variables collected included age, gender, and diagnosis, presenting symptom, radiation dose and fractionation, pre and post treatment grade of bleeding, pre and post treatment hemoglobin, toxicities, and the last follow up.

Response assessment was categorized into a complete response, partial response and no response. Qualitative variables were presented as frequencies and percentages. Descriptive analysis was applied for quantitative variables (mean, median, range and standard deviation). Demographic information is shown in the form of tables. A comparison was made for the bleeding scale before and after HRT using the Wilcoxon signed rank test. The comparison of mean hemoglobin levels before and after the HRT was calculated by paired t-test. Potential associations between bleeding control and different variables were estimated using Chi-square test. A p-value of less than 0.05 was considered as statistically significant. Data were analyzed with the Statistical Package for Social Sciences (SPSS) for Windows, version 22.0 IBM Corp., Armonk, NY.

## Results

Forty-two patients with advanced pelvic malignancies qualified for inclusion in the study after applying the inclusion/exclusion criteria. Among those analyzed, the median age was 67 years (range 37 – 95 years). The male and female proportion was 38% and 62% (n=16 and 26), respectively. Different cancers included uterine cancer 31% (n=13), cervical cancer 24% (n=10), bladder cancer 21% (n=9), rectal cancer 17% (n=7), and vulvar cancer in 7% (n=3) [Table 1]. The baseline WHO bleeding scale in these cases was found to be grade 1 in 12% (n=5), grade 2 in 55% (n=23) and grade 3 in 33% (n=14) cases. The median dose in our cohort was 20 Gy in five fractions over one week (range was 8 Gy to 40 Gy).

**Table 1:** Frequency distribution of diagnosis

Types of cancer	Total	%
Uterus	13	31%
Cervix	10	24%
Bladder	9	21%
Rectum	7	17%
Vulva	3	7%
Total	42	100%

Following HRT, the WHO bleeding score recorded at one-month post treatment was grade 0 in 57% (n = 24), grade 1 in 31% (n = 13), grade 2 in 7% (n = 3), grade 3 in 5% (n = 2) and grade 4 in none (n = 0) [Table 2]. Response rates were 57% complete response, 36% partial response and 7% no response.

**Table 2:** Frequency distribution of WHO bleeding grade at baseline and one-month post treatment WHO: World Health Organization

WHO Bleeding Grade	At baseline		At one month	
	n	%	n	%
G0	0	0	24	57%
G1	5	12%	13	31%
G2	23	55%	3	7%
G3	14	33%	2	5%
G4	0	0	0	0
Total	42	100%	42	100%

In the sub-category analysis, a satisfactory outcome assessment was performed. The study results showed that patients ≤60 years of age (n=18) achieved a satisfactory outcome in 78% cases (n=14) whereas patients >60 years of age (n=24) achieved a satisfactory outcome in 54% cases (n=13). Male patients (n=16) achieved a satisfactory outcome in 80% cases (n=13). Female patients (n=26) achieved satisfactory outcome in 73% cases (n=19). Similarly, the patients with the endometrial cervical (squamous cell) cancer were 10, in which satisfactory outcome was achieved in all cases (100%). Statistically, an insignificant difference was found between the age and gender with the outcome, and a statistically significant difference was found between the outcome with the type of cancer, i.e., p-values = 0.196 (age), 0.447 (gender), and 0.002 (cancer type) respectively [Table 3].

**Table 3:** Comparison of outcome with age, gender and type of diagnosis Satisfactory outcome = WHO Grade 0 or 1 bleeding at one-month post-RT *significant p-value (p <0.05) WHO: World Health Organization, RT: radiation therapy

		Satisfactory Outcome		Total	%	p-value
		No	Yes			
Age (years)	≤ 60	4	14	18	78%	0.196
	> 60	11	13	24	54%	
Gender	Male	3	13	16	81%	0.447
	Female	7	19	26	73%	
Type of cancer	Cervix	0	10	10	100%	0.002*
	Others	5	27	32	84%	

The mean hemoglobin level post-treatment versus mean hemoglobin level pre-treatment was found to be 9.6 g/dL versus 7.3 g/dL (p = 0.04) at one-month post-RT. Toxicity profile did not show any grade 3 or above acute toxicity in the study [Table 4].

**Table 4:** Descriptive statistics of hemoglobin at baseline and one-month post-treatment p-value for mean hemoglobin at baseline compared to that at one-month post-treatment *siginificant p-value (p <0.05) SD: standard deviation

Hemoglobin (g/dL)	At baseline	At one-month
Mean	7.34	9.62
SD	1.81	2.36
Minimum	5.66	7.41
Maximum	9.64	11.67
p-value	0.04*	

## Discussion

Bleeding occurs in approximately 6–10% of the patients with advanced cancer which is mainly attributed to the failure of local healing pathways. For the treating physician, it poses a clinical challenge as in some patients it may be the immediate cause of death. Pereira and Phan evaluated multiple modalities used in the management of hemorrhage in advanced malignancies [1]. Systemic treatments include blood products, vitamin K, vasopressin or desmopressin, somatostatin analogs, and antifibrinolytic agents. Local modalities include topical hemostatic agents, dressings, endoscopy, vessel ligation, tissue resection, cauterization, styptics, transcutaneous arterial embolization, or balloon placement [16]. Radiation therapy has been used as the first line of treatment for controlling active bleeding in tumors in cases where other local therapies have failed, thereby producing durable response rates [17-21]. A study by Biswal et al. [19] showed that HRT used in the management of bleeding carcinomas of the uterine cervix secured a bleeding-control rate of 100% within 48 hours following HRT, which appears to be in concordance with our study. Another study on gynecological cancers determining the hemostatic effect of RT was done by Kraiphibul et al. [22] in which 63% of cases of cancer-related bleeding were controlled within three fractions of HRT and 97% within five fractions of HRT. In our study, we assessed the treatment efficacy at one-month post-treatment and found out to have a 57% complete response in bleeding malignancies of the pelvic area.

Langendijk et al. [23] study on non-small cell lung cancer treated with HRT found a sufficient reduction in hemoptysis in 83% of cases. Similarly, Brundage et al. [24] have shown an 80% reduction in bleeding in non-small cell lung cancers. HRT has proven role in improving the subjective condition of bleeding patients. A published analysis by Lee et al. [20] reviewing 30 patients with gastric cancer bleeding found improvement in hemoglobin levels and decreased the need for blood transfusions in 91% of cases following treatment with HRT. In our cohort, we demonstrated a clinical response in terms of bleeding control with the mean hemoglobin scores improved at one-month follow up after HRT, i.e., 9.6 g/dL at one-month versus 7.3 g/dL at presentation (p = 0.04). Although in our study, we did not control for other hemostatic measures after delivery of HRT. Cihoric et al. [11] in their study reported a significant bleeding response in terms of control rates, 95% for uterovaginal lesions, 100% for the lung lesions and 90% for upper GI lesions at the end of HRT. This study remains one of the most extensive retrospective studies evaluating the role of HRT in different types of malignancies. In our study, we achieved 100% control rate in our cervical cancer patient subgroup, whereas other cancers also showed promising results regarding bleeding control and response assessment.

Various fractionation schedules (hypofractionated or conventional) in radiation therapy have been used to address the bleeding malignancies. In some studies, hypofractionated RT has been reported to be as effective as the standard fractionation regimens [23-24]. In patients with gastric cancer bleeding, dose fractionation regimen has ranged from an 8-Gy single fraction to 40 Gy in 16 fractions [17, 20, 25]. In a study by Asakura et al. [21], they used a schedule of 30 Gy in 10 fractions using a 6, 10 or 18-MV linear accelerator and found it to be adequate for palliation of advanced gastric cancer with bleeding. In our cohort, our median dose for controlling bleeding was 20 Gy delivered in five fractions over one week. We also used single fraction 8 – 10 Gy in cases with poor ECOG performance status and limited life expectancy. The second most common fractionation schedule in our study was 30 Gy in 10 fractions over two weeks.

A study by Mustafa et al. [26] described radiotherapy as a successful, time-efficient, cost-effective, and safe modality to alleviate the symptoms of cancer patients in their advanced stages. In their study the overall response rate after two weeks of completion of RT was 65%; the median follow-up of the patients was 109 days (range 7-280 days). The overall long-term symptom control was 20%. One study conducted by Srinivasan et al. [27] quoted the success rate of HRT up to 59% in advanced bladder cancer cases that presented with bleeding. Various studies have shown the hemostatic effectiveness of palliative radiotherapy in uterine and rectal cancer [28-29]. A study by Rasool et al. [30] concluded 88% response rate (n=22) in their study of 25 patients with a complete cessation of bleeding following HRT. They used two schedules, and both of the 15 Gy and 20 Gy dose schedules were equal in terms of efficacy. Treatment was well tolerated in both protocols without any significant adverse events. They concluded HRT to be a safe and effective option for controlling tumor bleeding. Similarly, our study did not reveal any grade 3 toxicity in our cohort. Our overall complete response rate was 57% and partial response in 36% cases. Our median dose delivered was 20 Gy, and we did not compare with other fractionation schedules. In our subgroup analysis, gender and age did not appear to have a statistically significant effect on the outcome, whereas the correlation of tumor type with the outcome was statistically significant (p = 0.002).  

The lack of data and limited available literature in this subject area warrants further investigation to evaluate and determine the factors that would be crucial in establishing the efficacy, safety and optimal dose in securing hemostasis in bleeding malignancies. This study is presumed to contribute to filling the gap in this area by adding the experience from a tertiary care institute from Saudi Arabia. Our limitations of the study include smaller sample size that restricted us randomization and controlled comparison to other fractionation regimens, analysis of limited types of tumors, shorter follow-up and not assessing the role of other hemostatic measures in the post-treatment period. A multi-institutional approach and a larger sample size, employing a control arm to compare the efficacy of different fractionation regimens would be an ideal study to assess the comparative efficacy and establish ideal fractionation regimen to attain hemostasis in this patient group.

## Conclusions

Hypofractionated radiotherapy appears to be a safe and effective non-invasive palliative treatment modality for securing hemostasis in advanced pelvic malignancy patients that presented with bleeding.
